# Effectiveness of adulticide and larvicide in controlling high densities of *Aedes aegypti* in urban environments

**DOI:** 10.1371/journal.pone.0246046

**Published:** 2021-01-25

**Authors:** André B. B. Wilke, Chalmers Vasquez, Augusto Carvajal, Monica Ramirez, Gabriel Cardenas, William D. Petrie, John C. Beier

**Affiliations:** 1 Department of Public Health Sciences, Miller School of Medicine, University of Miami, Miami, FL, United States of America; 2 Miami-Dade County Mosquito Control Division, Miami, FL, United States of America; Instituto Nacional de Salud Pública, MEXICO

## Abstract

Current management and control of *Aedes aegypti* populations in urban areas are based on the spraying of insecticides. Here, we evaluated the effectiveness of spraying larvicide (*Bacillus thuringiensis israelensis*) using a truck-mounted Buffalo Turbine and adulticide (Deltamethrin) using a Grizzly ULV Sprayer in an urban area with high densities of *Ae*. *aegypti* and many cryptic and difficult to reach aquatic breeding habitats. Experiments were conducted in a tire shop located in Miami-Dade County, Florida with approximately 100,000 used airplane tires. Insecticide interventions were performed after a baseline survey consisting of 3 weeks of collections, followed by two insecticide interventions: (i) application of the adulticide followed by the application of larvicide on the subsequent week; and (ii) application of both adulticide and larvicide on two consecutive weeks. The first insecticide intervention resulted in a non-significant decrease in the relative abundance of *Ae*. *aegypti*. On the other hand, the second insecticide intervention significantly reduced the *Ae*. *aegypti* relative abundance (*P* < 0.002). Our results demonstrated that the combined insecticide interventions on two consecutive weeks significantly reduced the relative abundance of *Ae*. *aegypti*. This result indicated that the larvicide was successfully propelled reaching cryptical and difficult to reach aquatic habitats. However, even though the number of mosquitoes was greatly reduced, it was still greatly above the 10-mosquito threshold by trap night used by the Miami-Dade Mosquito Control Division to deploy an inspector to survey the area. Considering the lack of new and effective mosquito control tools, efficient and mobile insecticide propellers such as Buffalo Turbine can be of great help to manage mosquito populations in urban areas.

## Introduction

Mosquito vectors are responsible for the transmission of many diseases [1]. Current estimates point to 390 million cases of dengue (DENV) per year [2]. The Zika virus (ZIKV) outbreak in the Americas between 2015 and 2018 had 1,003,509 confirmed cases [3], that lead to a significant increase in fetus malformation caused by ZIKV infection during pregnancy [4, 5]. Furthermore, reports of arbovirus outbreaks are becoming more frequent in previously non-endemic areas, such as the chikungunya (CHIKV) outbreaks in Europe [6–8]. As a consequence of the deterioration of the effectiveness of mosquito control strategies more than half of the world’s population is living in endemic areas [9].

*Aedes* (*Stegomyia*) *aegypti* is widely distributed around the globe [10]. It is the primary vector of CHIKV, DENV, yellow fever (YFV), and ZIKV [10–14], and is implied responsible for the majority of the cases worldwide. *Aedes aegypti* is remarkably adapted to thrive in urban and suburban areas alongside humans widely benefiting from anthropogenic alterations in the environment [15–20]. Being able to thrive in urban environments represent a significant advantage for *Ae*. *aegypti*. Only a few species are able to survive in the harsh urban environments and, for this reason, immature *Ae*. *aegypti* have more access to food and less crowded breeding habitats [21–23], as well as the virtual absence of natural predators and a high abundance of human hosts for blood feeding [24–27].

*Aedes aegypti* is considered an *r*-strategist (i.e., produces large offspring with a low probability of reaching adulthood) [28–30]. It oviposits an average of 100 eggs after each blood meal, potentially being able to produce more than 500 eggs during its lifetime. The lack of natural predators and larval competition makes it possible for many specimens to reach adulthood positively driving the population size [31, 32]. Large *Ae*. *aegypti* populations in urban areas most often require the employment of mosquito control strategies to artificially reduce its populations by, among other things, insecticide spraying. The combined use of larvicide and adulticide has been used in the attempt to control populations of vector mosquito species [33–36]. However, controlling *Ae*. *aegypti* populations in urban environments is problematic, relying on many critical steps that logically build on each other [37].

Furthermore, the indiscriminate use of insecticide for the control of mosquitoes has led to the development of resistance by *Ae*. *aegypti*, and has affected non-target insect populations such as bees and dragonflies [38–41]. Pyrethroids have been commonly used to control *Ae*. *aegypti* populations in urban areas. However, their effectiveness has been drastically reduced by the development of resistance by vector mosquito species [39, 42]. Even though the resistance to pyrethroids is widespread in *Ae*. *aegypti* populations it is not homogeneously distributed and can change over time [39, 43, 44]. *Bti* has been intensively used as a biocontrol tool to suppress populations of young instar mosquito vectors breeding in aquatic habitats. The intrinsic properties of the toxins produced by *Bti* naturally avoid the development of resistance by vector mosquitoes and are highly effective in killing mosquito vector species with minimum effect on non-target species [45]. However, *Bti* effectiveness can be drastically impaired by the presence of cryptic and difficult to reach aquatic habitats [46]. The side effects of insecticides in human populations have restricted even further the use of insecticides, with current control strategies prioritizing environmental ordinance and preventative mosquito control strategies other than reactive strategies based solely on insecticide spraying [47]. However, the use of insecticides is still one of the pillars of the Integrated Vector Management (IVM) to control high densities of *Ae*. *aegypti* in urban areas [48].

Effectively reducing a mosquito population using insecticides relies primarily on two factors: (i) an effective delivery method allowing the insecticide not only to reach the targeted mosquito population but to reach immature mosquitoes in cryptic aquatic habitats and hidden adult mosquitoes; and (ii) the use of an insecticide that effectively kills the mosquitoes and reduce the population size without the development of resistance by the target mosquito population. Truck-mounted Buffalo Turbine and Mist Sprayer Grizzly ULV Sprayer are highly mobile and capable of propelling a low volume spray of insecticide with a relatively constant droplet size with a maximum range of 200 meters [49]. Targeted and efficient insecticide spraying operations can be done using both the truck-mounted Buffalo Turbine and the Grizzly ULV Sprayer. On the other hand, aerial applications have to be done over larger areas requiring not only substantially more insecticide but also affecting non-target areas. The truck-mounted Buffalo Turbine can spray similar amounts of insecticide in a more controlled way.

The Buffalo Turbine is often used to spray the larvicide *Bacillus thurigiensis* var. *israelensis* (*Bti*). *Bti* natural toxins are highly toxic to mosquitoes and have minimal effect on non-target species [45]. Moreover, *Bti* toxins are naturally complex preventing the development of resistance by *Ae*. *aegypti* [45]. However, although there is consensus on the efficacy of *Bti* in controlling populations of immature mosquitoes, its effectiveness can be substantially reduced if it fails to be propelled in enough quantities in difficult to reach aquatic habitats, especially small containers, such as tires, gutters, bromeliads, flower pots, buckets, etc. or in aquatic habitats with partial access, such as sewage systems and storm drains [46, 50].

We evaluated the effectiveness of spraying the larvicide *Bti* and the adulticide Deltamethrin in an urban area with high densities of *Ae*. *aegypti*. Our primary objective was to determine the effectiveness of the Buffalo Turbine in propelling *Bti* and the Grizzly ULV Sprayer in propelling Deltamethrin to determine their efficacy in controlling *Ae*. *aegypti* in an area with many cryptic and difficult to reach aquatic habitats to help guide and improve mosquito control operations.

## Methods

### Study site

Tires have long been associated with the proliferation of vector mosquitoes [51–53]. Tires are particularly favorable habitats for the proliferation of mosquito vector species [54, 55]. Tires efficiently collect rainwater providing optimum conditions for immature mosquitoes by providing essential resources and protecting them from natural predators and the elements [56]. Motor vehicles, and therefore tires, are ubiquitous in urban and rural areas with current production ranging around 1.4 billion units every year. Discarded tires comprise a major challenge for public health due to the inherent difficulty in treating all potential microhabitats suitable for sustaining mosquito vector populations.

**Fig 1:**
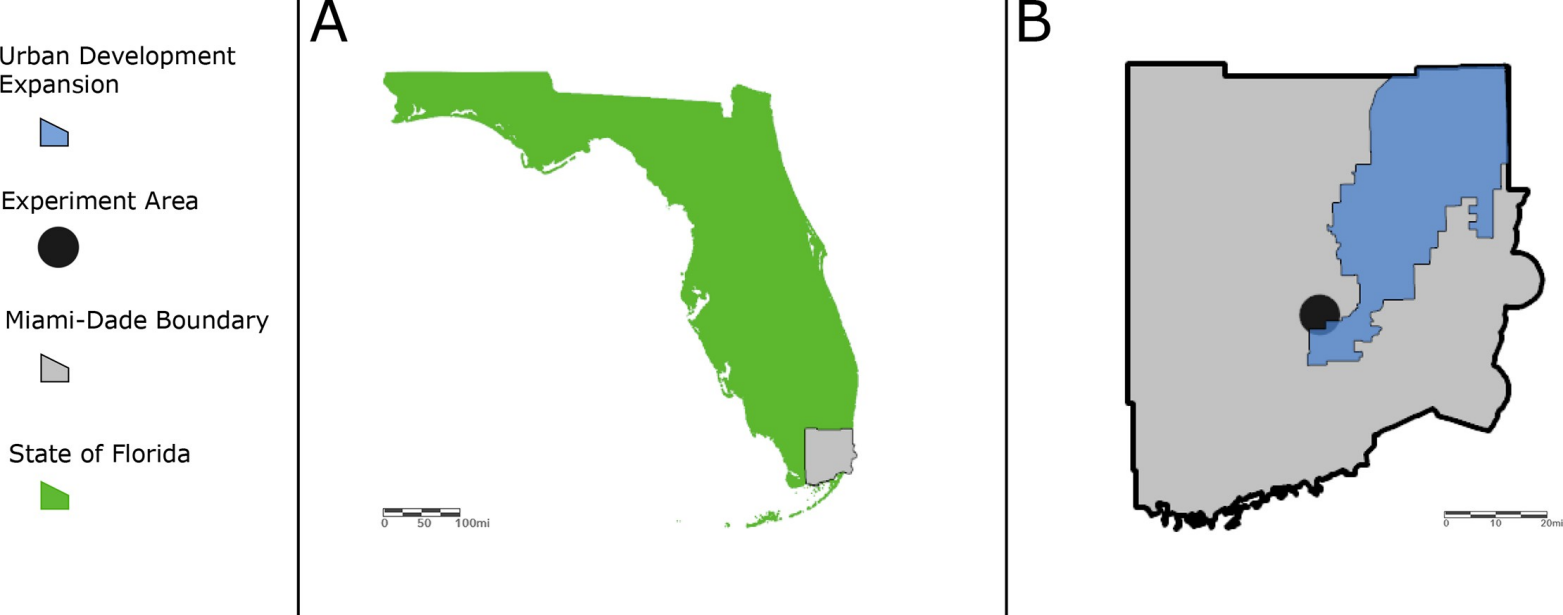
Maps displaying the approximate location of the tire shop in Miami-Dade County, Florida. (A) State of Florida; (B) Miami-Dade County. Coordinates: 25.775589, -80.196845.

For this reason, the experiments were performed in Miami-Dade County, Florida at a tire shop specialized in retreading aircraft tires located at the limits of the urban development expansion zone of Miami (Fig 1). This location was selected for having high densities of *Ae*. *aegypti* in a relatively small and constrained space, being notably extremely difficult to manage and control.

**Fig 2:**
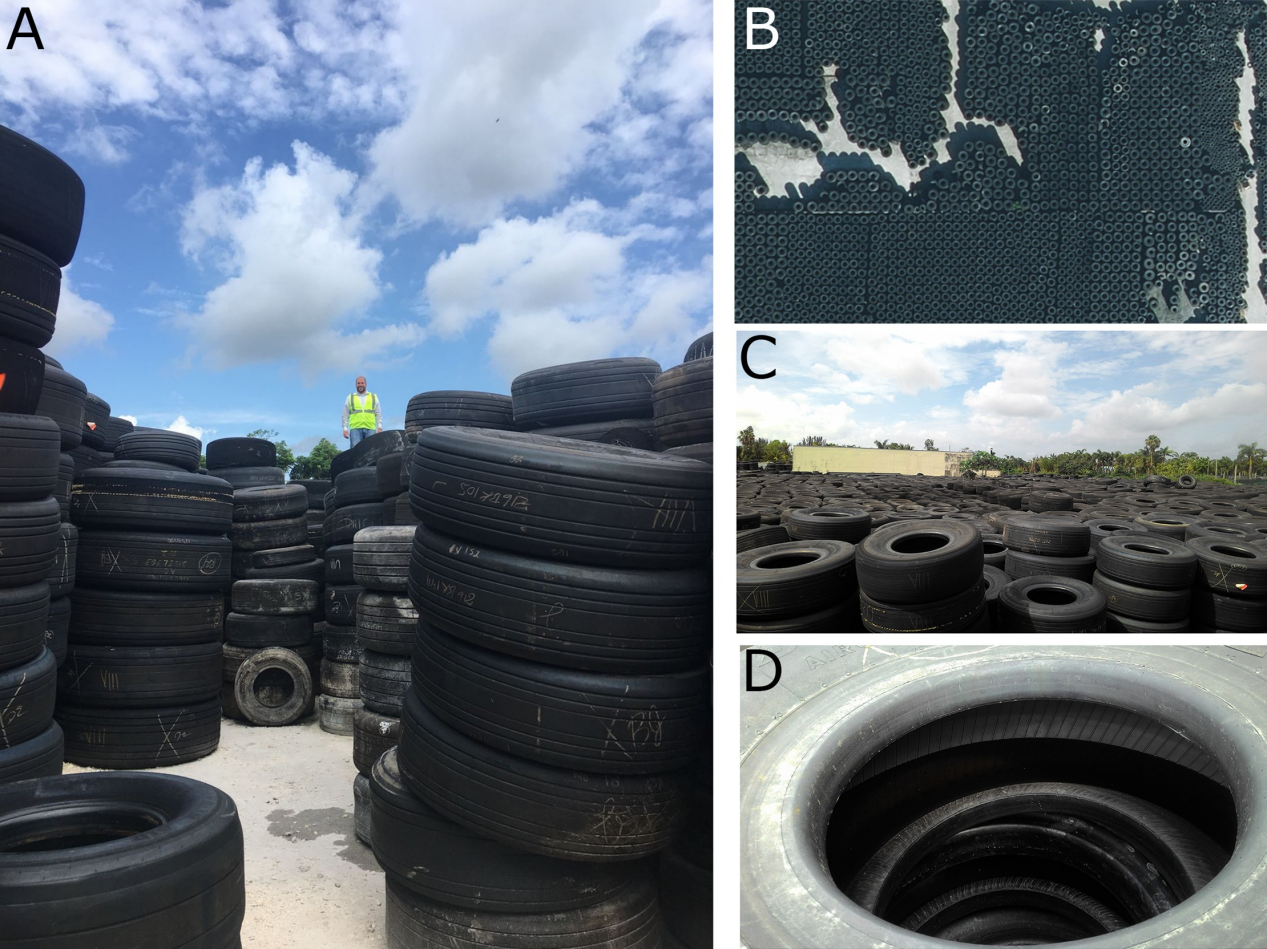
Tire shop environment. (A) Tire stacks; (B) aerial view of the tire shop; (C) view of the tire shop from the top of the tire stacks; and (D) tires filled with water organized in a stack.

The tire shop had approximately 100,000 used airplane tires organized in stacks ranging from 4 to 12 tires in a 3-acre property. The diameter of the tires ranged from 0.5 to 1.5 meters and weight from 12 to 150 kg. All tires were stored outdoors and therefore exposed to the elements including rainfall. As a result, the overwhelming majority of tires were filled with water throughout the duration of this study (Fig 2).

### Experimental design

We divided the tire shop and surrounding area into 6 areas to be able to compare the effectiveness of the Buffalo Turbine in propelling *Bti* and the Grizzly ULV Sprayer in propelling Deltamethrin in areas with different conditions and their effectiveness in reducing *Ae*. *aegypti* populations.

**Fig 3:**
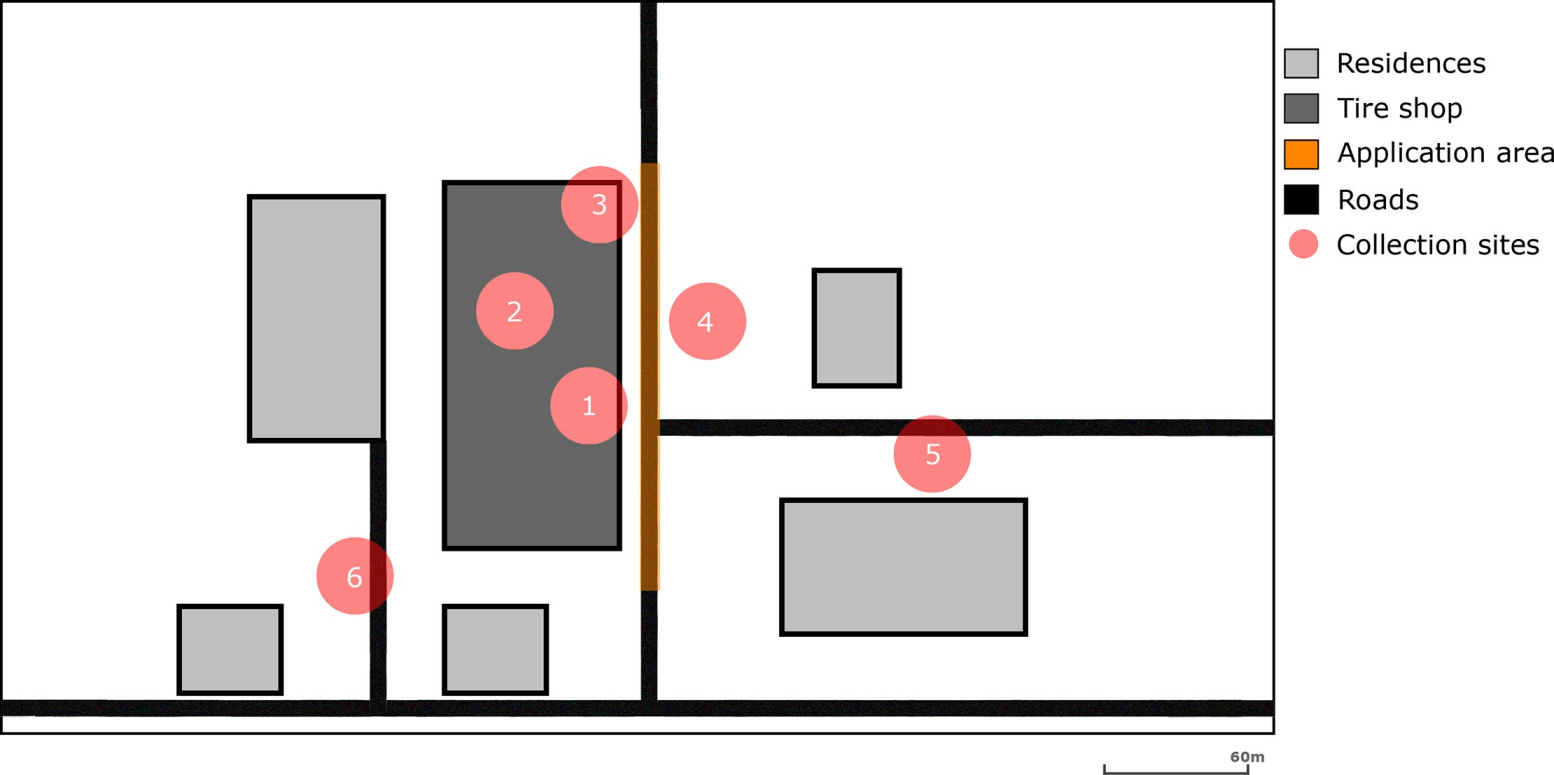
Map of the tire shop and surrounding area. (1) area near the office with higher flow of people; (2) virtual center of the tire shop with reduced presence of blood and sugar sources; (3) the northeastern corner of the property near vegetation; (4) bushed area across the property with many sugar sources from flowering plants and resting places; (5) abandoned railroad located at the back of the tire shop and near a bromeliad nursery with lots of vegetation; and (6) in front of a nearby residential house. The Buffalo Turbine and Grizzly ULV Sprayer spraying area is displayed in yellow.

Each testing area had unique features. Three areas inside the tire shop with identical size of 5 meters by 5 meters and 50 meters equidistant from each other: (1) area near the office with higher flow of people; (2) virtual center of the tire shop with reduced presence of blood and sugar sources; (3) the northeastern corner of the property near vegetation. Three areas outside the tire shop with identical size of 5 meters by 5 meters and more than 50 meters apart: (4) bushed area across the property with many sugar sources from flowering plants and resting places; (5) abandoned railroad located at the back of the tire shop and near a bromeliad nursery with many sugar sources; and (6) in front of a nearby residential house (Fig 3). All experiments were performed considering this experimental design.

### Immature and adult mosquito collections

Adult mosquitoes were collected weekly for 24 hours by 6 BG-Sentinel 2 traps (one in each of the 6 areas as described above) baited with CO_2_ [57] and deployed for the entire duration of the study (17 weeks) from June 7th to September 27th, 2018. Immature mosquitoes were collected on three different occasions: the first before any insecticide intervention (Week 3) and then on two occasions after each of the joint *Bti* and Deltamethrin insecticide interventions (Weeks 8 and 9). Collections of immatures were done in areas 1, 2 and 3. For each area, three tire stacks were taken outside using a forklift, then the water of each tire was drained using a shopvac, and the volume was measured and screened for immature mosquitoes. Larvae and pupae were collected with the aid of manual plastic pumps (turkey basters) and stored in plastic containers (100 ml) for transport. All collected mosquitoes were transported to the Miami-Dade County Mosquito Control Laboratory and subsequently morphologically identified using taxonomic keys [58].

### Insecticide interventions

Two commercial products were applied as aqueous solutions at the following label rates: Vectobac WDG (*Bti* 37.4% AI (Valent Biosciences, Libertyville, IL. Vectobac WDG was applied at a rate of ½ lb. per acre, using a Buffalo Turbine sprayer (Distributed Clarke, St. Charles IL). Vectobac WDG was mixed at a ratio of 2 lb. per gal. of water. The Ultra-Low Volume (ULV) adulticide applications were performed using DeltaGard (Deltamethrin 2% AI) (Bayer Environmental Science, Research Triangle Park, NC) diluted in water at a 1:1 ratio. Deltamethrin was applied each time at the label rate of 0.0009 lb., using a Grizzly ULV Sprayer (Clarke, St. Charles, IL) (S1 Table).

These insecticides are commonly used to control vector mosquitoes in urban environments. After the Zika virus outbreak in Miami-Dade County in 2016 [59], both *Bti* and Deltamethrin have been widely used by the Miami-Dade Mosquito Control Division to control *Ae*. *aegypti* populations in problematic urban habitats to help to prevent future outbreaks.

Insecticide interventions were performed after an initial baseline survey consisting of 3 weeks of collections (weeks 1 to 3), followed by two insecticide interventions: (i) application of the adulticide on week 4 and larvicide on week 5; and (ii) application of both adulticide and larvicide on week 7 and 8. Collections continued for the following 9 weeks until the mosquito population started to increase in numbers.

Insecticide application was done between 5 and 6 pm when the environmental conditions were more conducive for the propelling of *Bti* and Deltamethrin (S2 Table). Since insecticide application was done from the eastern part of the tire shop, winds blowing from the Southeastern direction were the most conducive for spraying the insecticides by propellers.

Because the study posed less than minimal risk to participants the Institutional Review Board at the University of Miami determined that the study was exempt from institutional review board assessment (IRB Protocol Number: 20161212).

### Data analysis

The sample data was divided into adult and larvae and pupae collection data. For the adult sample data, the sample was analyzed using a generalized linear mixed model using the log link and the negative binomial distribution for the count of mosquitoes as the dependent variable, with location and time as the independent variables. In Table 2, the six locations were averaged within the timepoint and the first 3 time points were averaged and used as the baseline level for timepoint comparison estimates. The overdispersion scale parameter was estimated, instead of being held constant at 1, and the Bonferroni used a correction value of 0.05/15 for the significance cutoff.

For the immature mosquito data, the modeling is similar but instead of average over the location within time, the locations had several tires sampled within location and timepoint. This enabled the analysis to be done as an interaction of location and time and the independent variables are time location and time by location. Therefore, in Tables 4 and 5, the dependent variable is larvae counts in Table 4 and pupae in Table 5, comparing time differences within location. They similarly had the scale parameter estimated and the Bonferroni correction labeled. Figures were produced using ArcGIS 10.2 (Esri, Redlands, CA) using freely available layers from the Miami-Dade County's Open Data Hub - https://gis-mdc.opendata.arcgis.com/

## Results

A total of 120,522 adult mosquitoes were collected during the study. However, even though the specimens collected were distributed among 11 species and 5 genera, *Ae*. *aegypti* comprised 99% of all collected mosquitoes. *Aedes aegypti* was the most abundant species in all six areas surveyed in this study. The community composition of species varied greatly presenting a lower species richness in the area comprising the tire shop (areas 1, 2, and 3) when compared to the surrounding area (areas 4, 5, and 6). Only five species have been collected inside the tire shop *Ae*. *aegypti*, *Aedes albopictus*, *Anopheles crucians*, *Culex nigripalpus*, and *Culex quinquefasciatus*. Whereas in the surrounding areas eleven species were collected *Aedes aegypti*, *Ae*. *albopictus*, *Aedes taeniorhynchus*, *An*. *crucians*, *Culex biscaynensis*, *Culex coronator*, *Culex interrogator*, *Cx*. *nigripalpus*, *Cx*. *quinquefasciatus*, *Psorophora columbiae*, and *Wyeomyia mitchellii*.

Results from the insecticide intervention after the initial 3 weeks baseline, consisting of spraying adulticide one week prior to the spraying of larvicide resulted in a decrease in the relative abundance of adult *Ae*. *aegypti*. However, the statistical analysis indicated that the reduction in the relative abundance of *Ae*. *aegypti* after the application of adulticide on week 4 was not significant when compared to the control baseline (*P* = 0.138). The application of larvicide on week 5 also had not had a significant effect on decreasing *Ae*. *aegypti* relative abundance yielding non-statistically significant values (*P* = 0.271).

**Fig 4:**
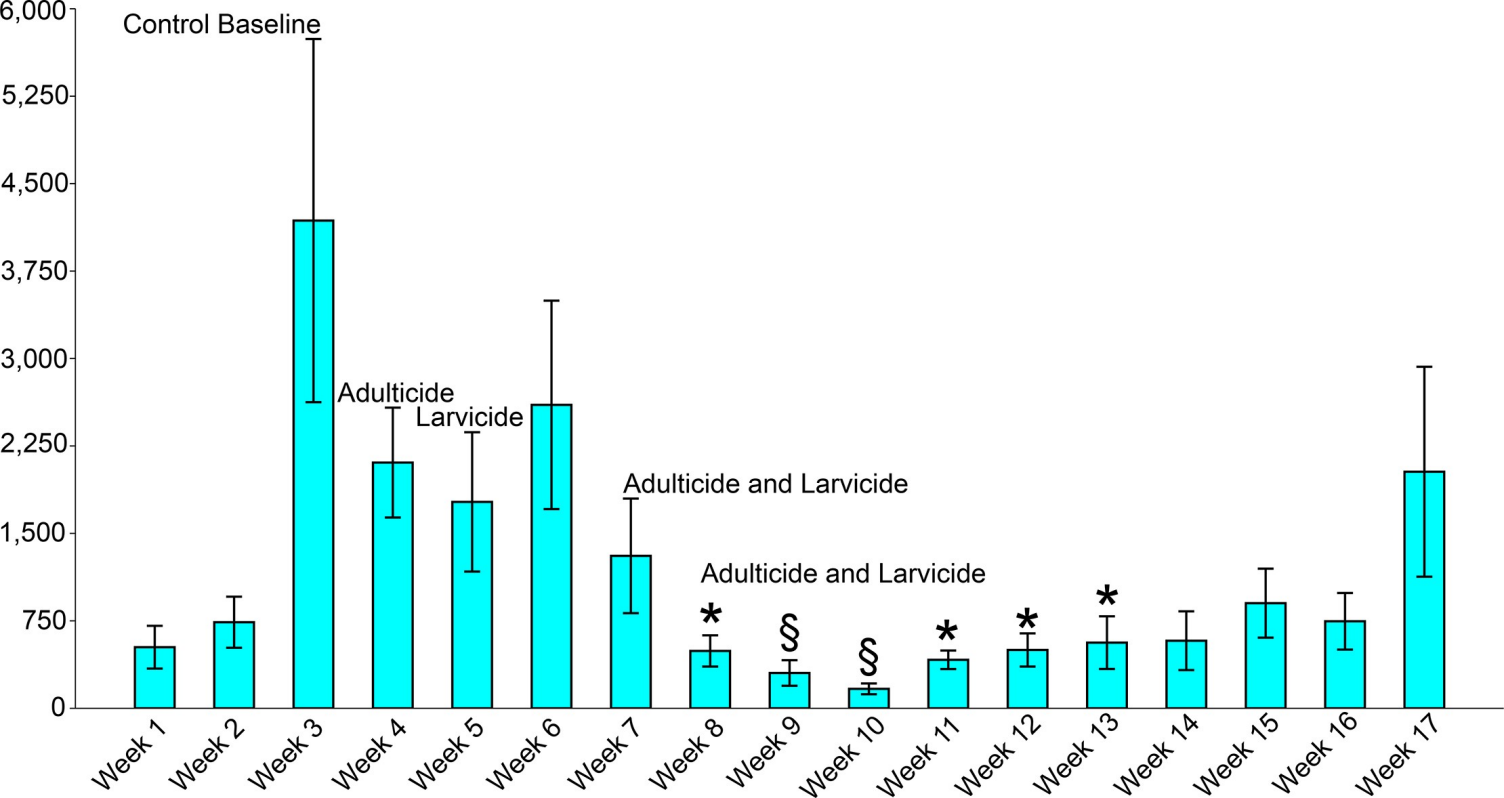
Bar chart displaying the effect of insecticide intervention in the abundance of adult *Ae*. *aegypti* at the study area in Miami-Dade County, Florida. Each bar displays the mean value; the whisker interval represents a 95% confidence interval standard error. Statistically significant values after multiple testing adjustment with Bonferroni. * = Significant values; § = Statistically significant values after multiple testing adjustment with Bonferroni.

We then moved on to two concomitant applications of both adulticide and larvicide. After the insecticide intervention, the relative abundance of *Ae*. *aegypti* was substantially reduced from 15,615 specimens collected on week 6 to 1,817 on week 9 and 1,001 on week 10, yielding highly significant values on weeks 9 and 10 (*P* = 0.002 and *P* < 0.0001, respectively) when compared to the control baseline. The effect of the insecticide intervention lasted until week 13 when the relative abundance of *Ae*. *aegypti* started to increase and the insecticide suppression effect was not statistically significant anymore. In week 17 the *Ae*. *aegypti* relative abundance reached the same level prior to the insecticide intervention (Tables 1 and 2, Fig 4).

**Table 1:** Effect of insecticide intervention in the abundance of adult mosquitoes at the study area in Miami-Dade County, Florida, from June 7th to September 27th, 2018.

Collection	*Aedes aegypti*	*Aedes albopictus*	*Aedes taeniorhynchus*	*Anopheles crucians*	*Culex biscaynensis*	*Culex coronator*	*Culex interrogator*	*Culex nigripalpus*	*Culex quinquefasciatus*	*Psorophora columbiae*	*Wyeomyia mitchelli*
# Week 1	3,145	3						1	11		
# Week 2	4,430	2						5	8		
# Week 3	25,098	149		1	2	11	1	27	16		1
* Week 4	12,646	70			9	2		4	7		
¥ Week 5	10,622	57						36			
Week 6	15,615	151	1					17	8		
§ Week 7	7,844	48				6				1	
§ Week 8	2,952	1									
Week 9	1,817	6		1		5		15	5		6
Week 10	1,001	4							4		2
Week 11	2,496	5				4		2	2		2
Week 12	3,002	3				5		2	2		
Week 13	3,379	3				3			1		2
Week 14	3,478	4				3		1	8		1
Week 15	5,410	31	1	2			28	7	42	4	
Week 16	4,478	4	4				14	5	8		
Week 17	12,174	2		1		3		16	20		3

# = Control Baseline

* = Adulticide

¥ = Larvicide

§ = Adulticide and Larvicide.

**Table 2:** Simple effect comparisons of median adult *Aedes aegypti* relative abundance per week using least squares means.

Comparisons	Estimate	Risk Ratio	*P*-Value
Baseline vs Week 4	-0.556	0.574	0.138
Baseline vs Week 5	-0.411	0.663	0.271
Baseline vs Week 6	-0.694	0.5	0.065
Baseline vs Week 7	-0.219	0.803	0.556
Baseline vs Week 8	0.918	2.504	**0.015**
Baseline vs Week 9	1.219	3.383	0.002*
Baseline vs Week 10	1.985	7.282	<0.0001*
Baseline vs Week 11	1.041	2.831	**0.006**
Baseline vs Week 12	0.879	2.408	**0.020**
Baseline vs Week 13	0.849	2.337	**0.025**
Baseline vs Week 14	0.659	1.934	0.079
Baseline vs Week 15	0.336	1.399	0.368
Baseline vs Week 16	0.445	1.56	0.234
Baseline vs Week 17	-0.573	0.564	0.126

Scale estimate was 0.3163; Used negative binomial with a log link. Significant values are in bold

*Statistically significant values after multiple testing adjustment with Bonferroni.

**Fig 5:**
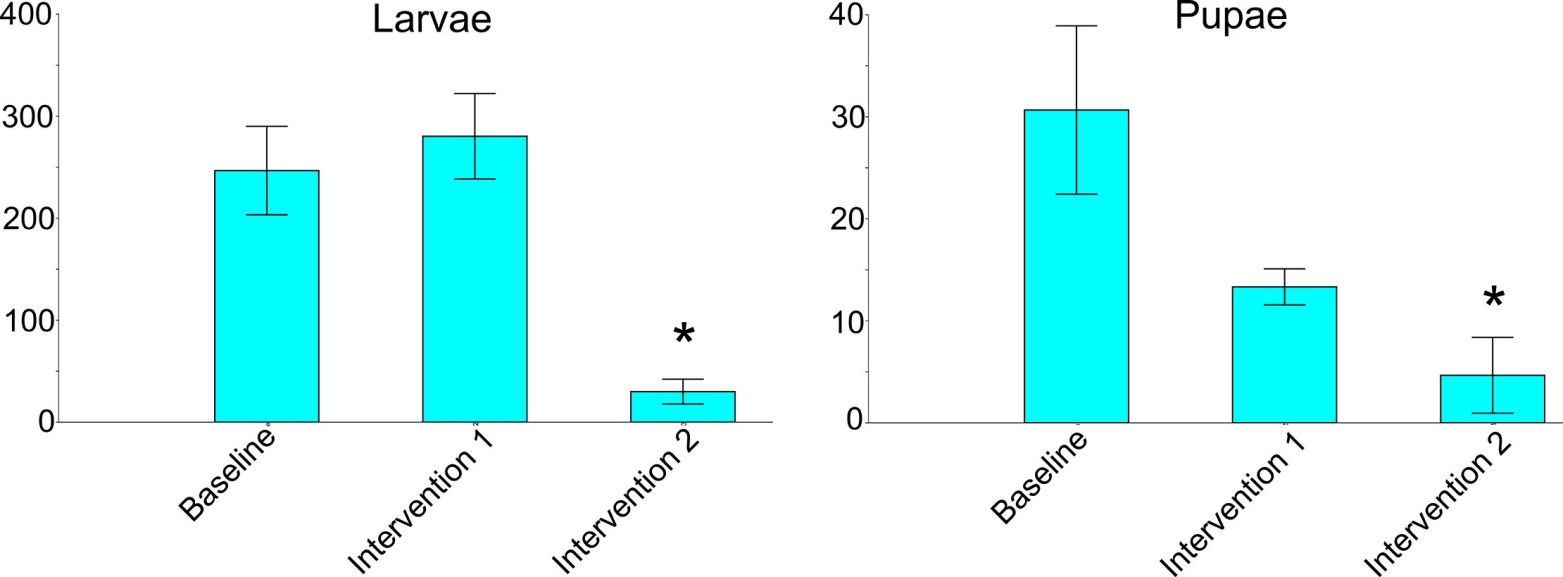
Bar chart displaying the effect of insecticide intervention in the abundance of immature *Ae*. *aegypti* at the study area in Miami-Dade County, Florida. Each bar displays the mean value; the whisker interval represents a 95% confidence interval standard error. * = Significant values.

The effectiveness of insecticide intervention 1 in reducing immature *Ae*. *aegypti* breeding in the tires was not significant when compared to the control baseline in none of the study areas. On the other hand, results from the second insecticide intervention significantly decreased the number of both larvae and pupae in all three study areas when compared both to the control baseline and to the first insecticide intervention. These results are indicating that the second insecticide intervention successfully decreased the number of immature *Ae*. *aegypti* being far more effective than the first insecticide intervention (Tables 3 and 4, and 5, Fig 5).

**Table 3:** Effect of insecticide intervention on the abundance of immature *Aedes aegypti* at the study area in Miami-Dade County, Florida, from June 7th to September 27th, 2018.

	Baseline (Week 3)		Intervention 1 (Week 8)		Intervention 2 (Week 9)	
Area	Larvae	Pupae	Larvae	Pupae	Larvae	Pupae
1	182	15	210	10	21	0
2	329	43	276	14	54	12
3	229	34	355	16	15	2

**Table 4:** Simple effect comparisons of median larvae *Aedes aegypti* densities per location per treatment using least squares means.

Location	Time	Adjusted Average Count/Tire Estimate	Denominator /Reference	Numerator	Adjusted Rate Ratio	*P*-value
Area 1	Baseline	10	Baseline	Treatment 1	1.044	0.9329
	Treatment 1	10	Baseline	Treatment 2	0.104	0.0001*
	Treatment 2	1	Treatment 1	Treatment 2	0.100	<0.0001*
Area 2	Baseline	16	Baseline	Treatment 1	0.724	0.5148
	Treatment 1	12	Baseline	Treatment 2	0.156	0.0004*
	Treatment 2	3	Treatment 1	Treatment 2	0.216	0.0027*
Area 3	Baseline	11	Baseline	Treatment 1	1.713	0.2898
	Treatment 1	19	Baseline	Treatment 2	0.06	<0.0001*
	Treatment 2	1	Treatment 1	Treatment 2	0.035	<0.0001*

Scale was 2.4936 with a standard error of 0.3346; Correlation between water volume and larvae was 0.211; Significant values (*P* < 0.05) are displayed in Bold

*Statistically significant values after multiple testing adjustment with Bonferroni.

**Table 5:** Simple effect comparisons of median pupae *Aedes aegypti* densities per location per treatment using least squares means.

Location	Time	Adjusted Average Count/Tire Estimate	Denominator /Reference	Numerator	Adjusted Rate Ratio	*P*-value
Area 1	Baseline	1	Baseline	Treatment 1	0.603	0.9329
	Treatment 1	0	Baseline	Treatment 2	0	0.0001*
	Treatment 2	0	Treatment 1	Treatment 2	0	<0.0001*
Area 2	Baseline	2	Baseline	Treatment 1	0.254	0.5148
	Treatment 1	1	Baseline	Treatment 2	0.266	0.0004*
	Treatment 2	1	Treatment 1	Treatment 2	1.048	0.0027*
Area 3	Baseline	2	Baseline	Treatment 1	0.52	0.2898
	Treatment 1	1	Baseline	Treatment 2	0.054	<0.0001*
	Treatment 2	0	Treatment 1	Treatment 2	0.103	<0.0001*

Scale was 3.0661 with a standard error of 0.7448; Correlation between water volume and pupae was 0.198; Significant values (*P* < 0.05) are displayed in Bold

*Statistically significant values after multiple testing adjustment with Bonferroni.

## Discussion

Our results demonstrated that combined insecticide interventions consisting of two consecutive applications of larvicide and adulticide using a truck-mounted Buffalo Turbine and Grizzly ULV Sprayer successfully reduced the relative abundance of *Ae*. *aegypti* for two weeks until the population started rebounding. This result indicated that the larvicide was successfully propelled by the Buffalo Turbine, reaching even the tires at the bottom of the stacks.

The Buffalo Turbine was an effective tool for propelling larvicide to control *Ae*. *aegypti* in difficult to reach aquatic habitats. After two consecutive applications at the tire shop the number of both immature and adult *Ae*. *aegypti* populations were significantly reduced when compared to the control baseline. However, the effectiveness of the combined insecticide intervention was directly associated with multiple and frequent applications and only had a significant effect in reducing the adult *Ae*. *aegypti* population for two weeks. Furthermore, even though the relative abundance of *Ae*. *aegypti* was greatly reduced, none of the insecticide interventions was able to reduce the *Ae*. *aegypti* population to acceptable levels. Even though the number of mosquitoes collected by the BG-Sentinel 2 traps was substantially reduced from 25,098 to 1,001 adult *Ae*. *aegypti*, it was still greatly above the 10-mosquito threshold by trap night used by the Miami-Dade Mosquito Control Division to deploy an inspector and initiate an insecticide intervention in the area.

The low residual activity of *Bti* observed here, being only significant for two weeks, may have been influenced by weather conditions commonly found in Miami such as elevated levels of rainfall. Of the 119 days of the study, it rained in 92, causing the tires to overflow and reducing the concentration of *Bti*, thus decreasing its effectiveness and residual activity (S2 Table) [60, 61]. Furthermore, the study site chosen for this study has an exceedingly large environmental carrying capacity allowing the proliferation of a considerable number of mosquitoes.

Considering that new strategies for controlling mosquito vectors based genetically modified mosquitoes are still a long way from being used operationally in the field [62], it is essential the development of effective mosquito management and control strategies based on the socioecological factors that are affecting their population dynamics within habitats across urban environments. The identification of environmental resources (e.g., larval habitats, suitable outdoor resting sites, sugar-feeding centers, and available hosts for blood-feeding) and modified urban features that contribute to the high concentrations of mosquito populations can be used for the development of preventative mosquito control efforts rather than relying only on less effective reactive mosquito control strategies, such as insecticide spraying.

Understanding how to control populations of vector mosquito species in problematic urban habitats that produce high numbers of vector mosquitoes, including construction sites, tire yards, bromeliad patches, urban farms, and cemeteries are key for the development of effective mosquito management and control strategies. Furthermore, determining how mosquito populations are affected when these problematic habitats lack key environmental resources necessary for mosquito survival, whereby mosquitoes must shift their basic ecology and behavior are likely to affect the effectiveness of mosquito management and control strategies.

Furthermore, the effectiveness of the Buffalo Turbine in longer application periods and different urban environments under real-world conditions is unknown. Such variations can lead to insecticide underdose or overdose with deleterious and undesired effects. Underdosing can lead to sub-optimal effects failing to significantly reduce the target population of vector mosquito species. On the other hand, overdosing can lead to toxic effects on non-target insect populations, as well as animals and humans [63–65].

Most of the chemical interventions done by the Miami-Dade Mosquito Control (and most Mosquito Control Districts in Florida, and the US) heavily rely on Buffalo Turbine to spray *Bti* and the Grizzly ULV to spray Deltamethrin. These are the most efficient insecticides with the lowest levels of resistance in *Ae*. *aegypti* population in Miami. Manually applying *Bti* granules or using a backpack insecticide sprayer to spray Deltamethrin in large areas such as in the one in this study is unfeasible and cannot be implemented in day-to-day control operations. Furthermore, both *Bti* and Deltamethrin have been extensively studied regarding their effectiveness, development of resistance by target species, toxicity, and more. Determining the effectiveness of the Buffalo Turbine in propelling *Bti* and the Grizzly ULV Sprayer in propelling Deltamethrin in controlling *Ae*. *aegypti* in an area with many cryptic and difficult to reach aquatic habitats is key to help guide and improve mosquito control operations. These cryptic and difficult to reach aquatic habitats are commonly found in urban areas and the effectiveness of a given insecticide application is mostly driven by the amount of insecticide that was propelled and reached those aquatic habitats in enough concentration to have the expected results.

Variations on droplet size may affect not only the insecticide spraying range but also the effectiveness in propelling it in enough concentrations to effectively reach and kill the target mosquito species. Mosquito surveillance also plays a major role in assessing the effectiveness of any insecticide intervention and should not be overlooked to help to guide and improve future mosquito management and control strategies.

## Conclusion

This study builds on the lessons learned during the ZIKV outbreak in Miami-Dade County, Florida and validates the current control efforts employed by the Miami-Dade Mosquito Control Division. Whereas preventative strategies should be the core of mosquito management and control, truck-mounted Buffalo Turbine sprayers can be used alongside Grizzly ULV Sprayer to effectively control populations of vector mosquito species in urban areas with many cryptic and difficult to reach habitats. Preventative spraying can also be a powerful tool to reduce mosquito populations in areas with high tourist concentrations and areas with increased outdoor activities with a higher risk for arbovirus transmission [66]. Considering the lack of new and effective mosquito control tools, an efficient and mobile *Bti* propeller such as Buffalo Turbines can be of great help to manage mosquito populations in urban areas.

## Supporting information

S1 Table Parameters and specifications of the insecticide applications. (DOCX)

S2 Table Climate variation in Miami-Dade County, Florida from September to October 2018. (DOCX)
