# Supplementary material for: Effectiveness of adulticide and larvicide in controlling high densities of Aedes aegypti in urban environments
Source: PLoS One. 2021 Jan 25;16(1):e0246046. doi: 10.1371/journal.pone.0246046 (PMC7833233; doi:10.1371/journal.pone.0246046)
Supplement: S1 Table — (DOCX) [file pone.0246046.s001.docx]

**S1 Table. Parameters and specifications of the insecticide applications.**

| **Parameter** | **Specification** |
| --- | --- |
| Flow Rate | 1.5 GPM |
| Vehicle Speed | 10 mph |
| Swath Width (SW) | 300 Feet |
| Average Vehicle Speed | 10 MPH |
| Tank MIX (2 lbs. of Vectobac WDG/gal of water) | 24% |
| Pounds of WDG/Acre | 0.5 lb/acre |
| Application Rate | 0.25 gal of mixture/acre |
| Pressure | 25 PSI |
| Blade Angle (MicronAir setting) | 55° |
| Droplet Size- VMD (Volume Median Diameter) | 120 µ |
