# Supplementary material for: Effectiveness of adulticide and larvicide in controlling high densities of Aedes aegypti in urban environments
Source: PLoS One. 2021 Jan 25;16(1):e0246046. doi: 10.1371/journal.pone.0246046 (PMC7833233; doi:10.1371/journal.pone.0246046)
Supplement: S2 Table — (DOCX) [file pone.0246046.s002.docx]

**S2 Table. Climate variation in Miami-Dade County, Florida from September to October 2018.**

| Date | Max Temp | Min Temp | Avg Temp | Precipitation | Wind Speed (mph) | Wind Direction |
| --- | --- | --- | --- | --- | --- | --- |
| 6/1/2018 | 86 | 75 | 80.5 | 0 |  |  |
| 6/2/2018 | 89 | 74 | 81.5 | 0 |  |  |
| 6/3/2018 | 92 | 77 | 84.5 | T |  |  |
| 6/4/2018 | 92 | 78 | 85 | 0 |  |  |
| 6/5/2018 | 93 | 79 | 86 | 0 |  |  |
| 6/6/2018 | 94 | 77 | 85.5 | T |  |  |
| 6/7/2018 | 89 | 71 | 80 | 1.36 |  |  |
| 6/8/2018 | 88 | 76 | 82 | 0.01 |  |  |
| 6/9/2018 | 86 | 71 | 78.5 | 2.37 |  |  |
| 6/10/2018 | 85 | 76 | 80.5 | 0.01 |  |  |
| 6/11/2018 | 85 | 75 | 80 | 0.05 |  |  |
| 6/12/2018 | 87 | 75 | 81 | 0.13 |  |  |
| 6/13/2018 | 88 | 77 | 82.5 | 0 |  |  |
| 6/14/2018 | 88 | 73 | 80.5 | 1.43 |  |  |
| 6/15/2018 | 89 | 74 | 81.5 | 0.02 |  |  |
| 6/16/2018 | 89 | 74 | 81.5 | 0.02 |  |  |
| 6/17/2018 | 90 | 77 | 83.5 | T |  |  |
| 6/18/2018 | 89 | 77 | 83 | 0 |  |  |
| 6/19/2018 | 88 | 78 | 83 | 0 |  |  |
| 6/20/2018 | 92 | 77 | 84.5 | 0 |  |  |
| 6/21/2018 | 94 | 78 | 86 | 0.04 |  |  |
| 6/22/2018 | 94 | 75 | 84.5 | 0.43 |  |  |
| 6/23/2018 | 91 | 74 | 82.5 | 0.18 |  |  |
| 6/24/2018 | 90 | 76 | 83 | 0.76 |  |  |
| 6/25/2018 | 90 | 78 | 84 | 0 |  |  |
| 6/26/2018 | 90 | 78 | 84 | 0.03 |  |  |
| 6/27/2018 | 89 | 75 | 82 | 0.27 |  |  |
| 6/28/2018 | 92 | 75 | 83.5 | 0.14 | 5 | WSW |
| 6/29/2018 | 90 | 75 | 82.5 | T |  |  |
| 6/30/2018 | 87 | 75 | 81 | 0.64 |  |  |
| 7/1/2018 | 90 | 75 | 82.5 | 0.06 |  |  |
| 7/2/2018 | 91 | 78 | 84.5 | 0 |  |  |
| 7/3/2018 | 89 | 78 | 83.5 | 0.33 |  |  |
| 7/4/2018 | 89 | 74 | 81.5 | 0.06 |  |  |
| 7/5/2018 | 90 | 76 | 83 | 0.07 | 4.35 | SE |
| 7/6/2018 | 90 | 72 | 81 | 0.67 |  |  |
| 7/7/2018 | 90 | 75 | 82.5 | 0.72 |  |  |
| 7/8/2018 | 91 | 78 | 84.5 | 0 |  |  |
| 7/9/2018 | 92 | 79 | 85.5 | 0 |  |  |
| 7/10/2018 | 90 | 74 | 82 | 0.45 |  |  |
| 7/11/2018 | 90 | 75 | 82.5 | 0.04 |  |  |
| 7/12/2018 | 91 | 75 | 83 | 0.1 |  |  |
| 7/13/2018 | 93 | 75 | 84 | 0.1 |  |  |
| 7/14/2018 | 91 | 77 | 84 | 0 |  |  |
| 7/15/2018 | 91 | 76 | 83.5 | 0 |  |  |
| 7/16/2018 | 91 | 80 | 85.5 | 0 |  |  |
| 7/17/2018 | 92 | 79 | 85.5 | T |  |  |
| 7/18/2018 | 93 | 76 | 84.5 | 0.41 |  |  |
| 7/19/2018 | 93 | 79 | 86 | 0.07 | 5.5 | SSE |
| 7/20/2018 | 92 | 78 | 85 | 0.34 |  |  |
| 7/21/2018 | 94 | 79 | 86.5 | T |  |  |
| 7/22/2018 | 93 | 79 | 86 | 0.05 |  |  |
| 7/23/2018 | 94 | 75 | 84.5 | 0.3 |  |  |
| 7/24/2018 | 88 | 75 | 81.5 | 1.38 |  |  |
| 7/25/2018 | 92 | 79 | 85.5 | T |  |  |
| 7/26/2018 | 92 | 76 | 84 | 0.03 | 5.5 | S |
| 7/27/2018 | 90 | 76 | 83 | T |  |  |
| 7/28/2018 | 93 | 74 | 83.5 | 2.15 |  |  |
| 7/29/2018 | 87 | 75 | 81 | 0.36 |  |  |
| 7/30/2018 | 85 | 75 | 80 | 0.22 |  |  |
| 7/31/2018 | 90 | 77 | 83.5 | 0.11 |  |  |
| 8/1/2018 | 90 | 78 | 84 | 0.11 |  |  |
| 8/2/2018 | 90 | 74 | 82 | 0.38 |  |  |
| 8/3/2018 | 90 | 78 | 84 | 0.31 |  |  |
| 8/4/2018 | 90 | 76 | 83 | 0.08 |  |  |
| 8/5/2018 | 90 | 80 | 85 | 0.03 |  |  |
| 8/6/2018 | 91 | 76 | 83.5 | 0.12 |  |  |
| 8/7/2018 | 90 | 79 | 84.5 | 0.05 |  |  |
| 8/8/2018 | 90 | 79 | 84.5 | 0 |  |  |
| 8/9/2018 | 92 | 73 | 82.5 | 0.72 |  |  |
| 8/10/2018 | 94 | 73 | 83.5 | 1.36 |  |  |
| 8/11/2018 | 91 | 74 | 82.5 | 2.17 |  |  |
| 8/12/2018 | 88 | 74 | 81 | 0.26 |  |  |
| 8/13/2018 | 89 | 75 | 82 | 0.53 |  |  |
| 8/14/2018 | 90 | 79 | 84.5 | T |  |  |
| 8/15/2018 | 91 | 80 | 85.5 | 0 |  |  |
| 8/16/2018 | 90 | 79 | 84.5 | T |  |  |
| 8/17/2018 | 91 | 81 | 86 | T |  |  |
| 8/18/2018 | 90 | 76 | 83 | 0.72 |  |  |
| 8/19/2018 | 90 | 76 | 83 | T |  |  |
| 8/20/2018 | 91 | 79 | 85 | T |  |  |
| 8/21/2018 | 90 | 78 | 84 | 0 |  |  |
| 8/22/2018 | 90 | 80 | 85 | 0.57 |  |  |
| 8/23/2018 | 90 | 79 | 84.5 | T |  |  |
| 8/24/2018 | 90 | 75 | 82.5 | 0.81 |  |  |
| 8/25/2018 | 88 | 73 | 80.5 | 0.26 |  |  |
| 8/26/2018 | 90 | 75 | 82.5 | 0.22 |  |  |
| 8/27/2018 | 90 | 76 | 83 | 0.54 |  |  |
| 8/28/2018 | 89 | 76 | 82.5 | 0.22 |  |  |
| 8/29/2018 | 89 | 78 | 83.5 | 0.03 |  |  |
| 8/30/2018 | 87 | 78 | 82.5 | 0.09 |  |  |
| 8/31/2018 | 89 | 79 | 84 | T |  |  |
| 9/1/2018 | 89 | 76 | 82.5 | 0.56 |  |  |
| 9/2/2018 | 87 | 75 | 81 | 0.25 |  |  |
| 9/3/2018 | 81 | 74 | 77.5 | 2.53 |  |  |
| 9/4/2018 | 89 | 77 | 83 | 0.06 |  |  |
| 9/5/2018 | 89 | 79 | 84 | 0.01 |  |  |
| 9/6/2018 | 88 | 77 | 82.5 | 0.05 |  |  |
| 9/7/2018 | 85 | 74 | 79.5 | 0.28 |  |  |
| 9/8/2018 | 85 | 73 | 79 | 0.13 |  |  |
| 9/9/2018 | 89 | 75 | 82 | 1.01 |  |  |
| 9/10/2018 | 86 | 75 | 80.5 | 0 |  |  |
| 9/11/2018 | 89 | 79 | 84 | 0 |  |  |
| 9/12/2018 | 92 | 79 | 85.5 | 0 |  |  |
| 9/13/2018 | 92 | 79 | 85.5 | 0 |  |  |
| 9/14/2018 | 90 | 77 | 83.5 | 0 |  |  |
| 9/15/2018 | 91 | 75 | 83 | 2.02 |  |  |
| 9/16/2018 | 90 | 78 | 84 | 0 |  |  |
| 9/17/2018 | 91 | 81 | 86 | 0 |  |  |
| 9/18/2018 | 90 | 79 | 84.5 | 0.06 |  |  |
| 9/19/2018 | 90 | 79 | 84.5 | 0 |  |  |
| 9/20/2018 | 91 | 78 | 84.5 | T |  |  |
| 9/21/2018 | 90 | 73 | 81.5 | 0.55 |  |  |
| 9/22/2018 | 89 | 76 | 82.5 | 0.04 |  |  |
| 9/23/2018 | 90 | 78 | 84 | 0 |  |  |
| 9/24/2018 | 91 | 80 | 85.5 | 0.05 |  |  |
| 9/25/2018 | 90 | 77 | 83.5 | T |  |  |
| 9/26/2018 | 91 | 81 | 86 | T |  |  |
| 9/27/2018 | 90 | 82 | 86 | T |  |  |
